# P-263. Informing Multi-level HIV Prevention Strategies Through Molecular Network Analysis in Georgia

**DOI:** 10.1093/ofid/ofaf695.484

**Published:** 2026-01-11

**Authors:** Carlos s Saldana, Olakunle Ogunbayo, Aimee Graciela Rivera-Solis, Adaiah Soibi-Harry, Masonia Traylor, Claudia E Ordóñez, Shirin Jabbarzadeh, Juan D Patino-Mateus, Santiago Ramon, Amalia Aldredge, Jenna Gettings, Daniel Mauck, Kirsten Oliver, Latasha Terry, Jane Y Scott

**Affiliations:** Emory University School of Medicine, Atlanta, GA; Emory Rollins School of Public Health, Atlanta, Georgia; Emory University School of Medicine, Atlanta, GA; Emory University, Atlanta, Georgia; Lady Burgandy, Atlanta, Georgia; Emory University, Atlanta, Georgia; Emory University School of Medicine, Atlanta, GA; Emory University School of Medicine, Atlanta, GA; Emory University School of Medicine, Atlanta, GA; Emory University, Atlanta, Georgia; Centers for Disease Control and Prevention, Atlanta, Georgia; Georgia Department of Public Health, Atlanta, Georgia; Georgia Department of Public Health, Atlanta, Georgia; Georgia Department of Public Healtb, Atlanta, Georgia; Emory University School of Medicine, Fulton County Board of Health, Atlanta, Georgia

## Abstract

**Background:**

Georgia remains heavily impacted by HIV, particularly among Black and Latino communities. HIV molecular epidemiology (HME) uses HIV pol gene sequence similarity to detect rapidly transmitting networks in near real time, supporting timely public health response and resource allocation. However, integration of behavioral, sociodemographic, and network-level dynamics into HME-informed interventions remains limited. To address this, we developed epidemiologic profiles of five HIV molecular networks in Georgia and applied a multi-level framework to guide equitable, data-driven public health action.

**Table 1.**
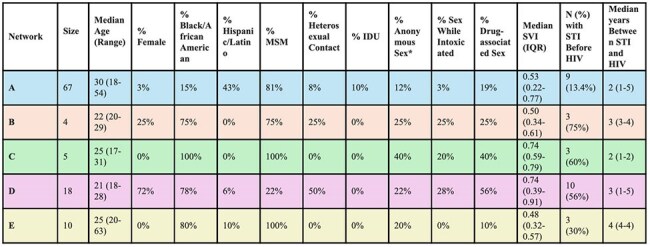
Demographic, Behavioral, Structural, and Prior STI Characteristics of Individuals in Five HIV Molecular Networks in Georgia. MSM = Men who have sex with men. IDU = Injection drug use. SVI = Social Vulnerability Index (range: 0–1), matched to eHARS data by census tract at time of HIV diagnosis, with higher values indicating greater community-level vulnerability. “Anonymous Sex”: refers to sexual activity with a partner whose identity is unknown. “Drug-associated sex” refers to sex with a partner who injects drugs, sex exchanged for drugs, or sex under the influence of injection drug use. “STI Before HIV” indicates individuals with a documented STI diagnosis prior to their HIV diagnosis. Median and range reflect the number of years between earliest recorded STI and HIV diagnosis.

**Table 2.**
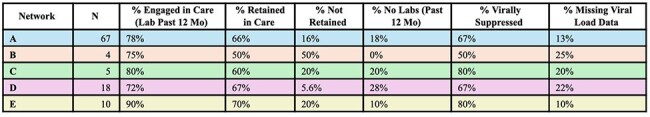
Care Continuum Outcomes Among Individuals in Five HIV Molecular Networks in Georgia.

Care engagement and retention were assessed using HIV viral load data from surveillance systems. Individuals were considered engaged if they had ≥1 lab in the past 12 months and retained if they had ≥2 labs ≥3 months apart. Viral suppression was defined as the most recent viral load <200 copies/mL.

**Methods:**

We analyzed five molecular networks using 2024 surveillance data from the Georgia Department of Public Health, including HIV-TRACE (molecular data), eHARS (HIV surveillance), and SendSS (Georgia’s notifiable disease reporting). We examined demographic, clinical, behavioral, and structural characteristics, including Social Vulnerability Index (SVI) scores. Data from 31 named partners were also assessed to identify prevention gaps.

**Table 3.**
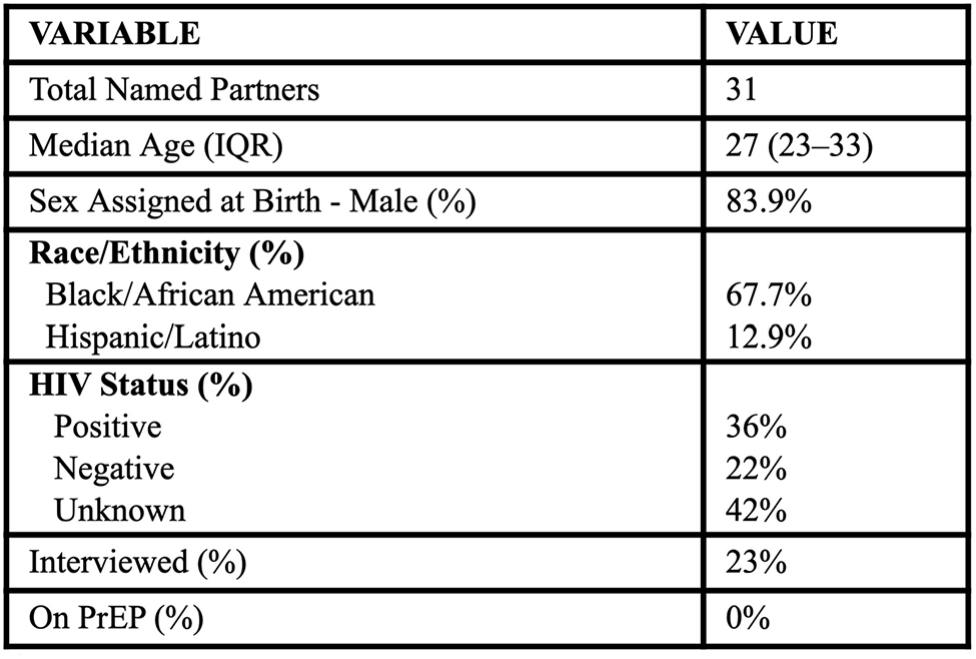
Demographic and Clinical Characteristics of Named Partners Linked to Five HIV Molecular Networks in Georgia. Data represents 31 individuals named through partner services associated with network members. HIV status reflects the most recent available surveillance data. “Interview” indicates whether named partners participated in interviews. “On PrEP” use was based on recorded referral or reported uptake.

**Table 4.**
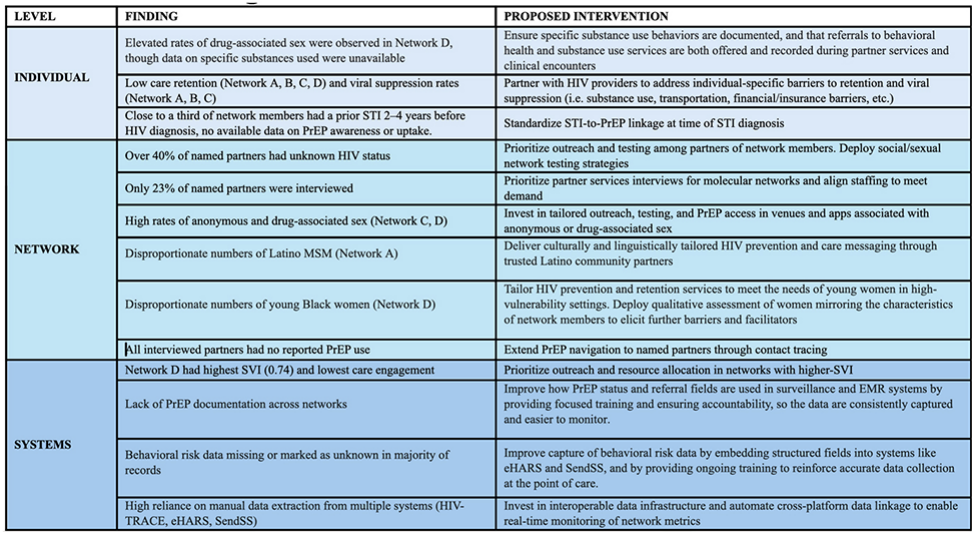
Opportunities for Intervention at the Individual, Network, and Systems Levels Based on Findings from Five HIV Molecular Networks and Named Partners in Georgia.

**Results:**

Among 104 individuals across five networks (range: 4–67), profiles varied by composition and risk (Table 1). Network A included 43% Latino men (median age 30, SVI 0.53); Network D was mostly (72%) young Black women (median age 21, SVI 0.74) with high behavioral risk (56% drug-associated sex, 28% intoxicated sex, 22% anonymous sex). Over 99% of records lacked key behavioral data, including substance use and PrEP linkage. Prior STIs were recorded in 27%, with a median lead time of 2–4 years before HIV diagnosis. Care engagement ranged from 67–90%, with lower retention and viral suppression in higher-SVI networks (Table 2). Among named partners, 36% were HIV-positive, 42% had unknown status, only 23% were interviewed by health departments, and none were on PrEP (Table 3).

**Conclusion:**

Findings highlight missed opportunities for prevention and care in fast-growing networks. We propose a multi-level response framework (Table 4) aligned with CDC guidance to improve PrEP access, retention, partner services, and data integration.

**Disclosures:**

All Authors: No reported disclosures

